# State of the Art on Thyroid Cancer Biology and Oncology

**DOI:** 10.3390/biomedicines14010168

**Published:** 2026-01-13

**Authors:** Federica Vaio, Camilla Moliterni, Stefania Mardente, Roberta Misasi, Emanuela Mari

**Affiliations:** 1 Department of Experimental Medicine, Sapienza University of Rome, Viale Regina Elena 324, 00161 Rome, Italy; federica.vaio@uniroma1.it (F.V.); c.moliterni@uniroma1.it (C.M.); roberta.misasi@uniroma1.it (R.M.); 2 Department of Life Science, Health and Health Professions, Link Campus University, Via del Casale di San Pio V 44, 00165 Rome, Italy; e.mari@unilink.it

**Keywords:** WHO 5th Classification (2022), *MAPK/ERK* and *PI3K/AKT/mTOR* pathways, *BRAF-V600E* and *TERT* promoter mutations, *RAS* mutations, mtDNA, microRNAs, personalized treatment, tumor microenvironment

## Abstract

Thyroid cancer (TC) incidence is rising, necessitating a refined understanding of its complex biology, particularly for advanced forms. This review synthesizes the state-of-the-art knowledge, guided by the WHO 5th Classification (2022), which incorporates molecular findings and introduces categories like Differentiated High-Grade Thyroid Carcinoma (DHGTC) to better stratify prognosis. The review summarizes the molecular changes in thyroid cancer (TC) by establishing a clear link between specific oncogenic alterations and the resulting tumor phenotype, prognosis, risk stratification and therapeutic vulnerabilities. The central importance of the review lies in its comprehensive integration of these molecular changes with the resulting immunological microenvironment and the rationale for novel, personalized therapies. Moreover, high-level genomic instability within aggressive thyroid malignancies promotes an immunosuppressive tumor microenvironment via the selection and recruitment of suppressive immune components, contributing to immune evasion and poor prognosis. This characteristic immunosuppression identifies the aggressive tumors as prime candidates for targeted immunotherapies. The review implicitly argues that understanding the molecular drivers of this immunosuppression is essential for designing effective clinical trials using these novel agents. Diagnostic advancements, including molecular testing for high-risk mutations (*BRAF*, *TERT*) and the integration of Artificial Intelligence (AI) for refined risk stratification, are enabling personalized treatment. The evolving molecular and clinical understanding allows for a paradigm shift toward individualized therapies that balance optimal disease control with minimizing morbidity, especially in the context of high-risk disease.

## 1. Introduction

Thyroid cancer, while often having a favorable prognosis, presents a complex and evolving landscape in terms of its biology and oncology. Recent advancements have significantly deepened our understanding of its molecular underpinnings and opened new avenues for diagnosis and treatment, particularly for advanced and aggressive forms [1]. Incidence of thyroid cancer of follicular cell derivation has risen considerably over the past few decades, largely attributed to increased detection through widespread imaging and fine-needle aspiration biopsies [2,3,4]. While many newly diagnosed cases are indolent, there is also a noted increase in advanced-stage disease. Overall, thyroid cancer has an excellent 5-year relative survival rate (around 98.5%). However, this varies significantly by histological subtype and disease stage. In this review we will refer to the 5th edition of the WHO Classification of Endocrine and Neuroendocrine Tumors (2022) [5] as it reflects tumor biology, incorporates molecular findings, and improves prognostication. The tumors are classified based on the cell of origin (Follicular cells or C-cells) and further categorized by their prognostic risk (Benign, Low-Risk, Malignant). A grading system is also introduced in the new classification for previously considered “well-differentiated” thyroid carcinomas, recognizing that some differentiated tumors behave more aggressively. The new category called Differentiated High-Grade Thyroid Carcinoma (DHGTC) includes Papillary Thyroid Carcinomas (PTC), Follicular Thyroid Carcinomas (FTC), and Oncocytic Carcinomas (OC) that retain their differentiated morphology but possess high-grade features (i.e., high mitotic index and necrosis) with a prognosis similar to Poorly Differentiated Thyroid Carcinoma (PDTC) (Table 1).

## 2. Molecular Alterations in the Different Subtypes of Thyroid Cancer

The concept of clinical aggressiveness in thyroid oncology is related more to the activation of universal signaling pathways than to their histological appearance. Rather than each subtype possessing a unique genetic signature, different thyroid cancers—ranging from papillary to anaplastic—often share common oncogenic features that function as central engines for growth and when they are mutated, drive cellular proliferation and invasion regardless of the tumor’s origin. This shift in perspective means that researchers and clinicians are increasingly focusing on the functional impact of these molecular drivers, viewing thyroid cancer as a spectrum of shared signaling disruptions where the intensity and combination of alterations dictate how aggressively the disease will behave.

### 2.1. TERT Promoter Alterations: The Aggressiveness Amplifier

The Telomerase Reverse Transcriptase (TERT) promoter mutation is a distinct, powerful prognostic marker. TERT is the catalytic subunit of telomerase, and its upregulation grants cancer cells replicative immortality [6]. The most common specific mutation sites are point mutations at C228T and C250T within the TERT promoter that create new binding sites for transcription factors, most notably the ETS family (such as GABPA and GABPB1) that promote telomere maintenance and elongation, conferring immortality and unlimited proliferative potential [7].

### 2.2. The Synergistic BRAF/TERT Co-Mutation

The most critical finding is the co-occurrence of the BRAF-V600E mutation and a TERT promoter mutation. This genetic combination defines the most aggressive subset of PTC patients, exhibiting the highest rates of distant metastasis, progression to poorly differentiated cancer, disease-specific mortality and resistance to radioiodine. This synergistic effect makes molecular testing for both BRAF and TERT mutations a crucial tool in modern risk stratification for PTC [8]. TERT hyperactivation contributes to immunosuppressive modulation of the Tumor Microenvironment (TME) by not only driving tumor growth but also enabling immune evasion [9]. This oncogenic activation is connected to inflammation and immunosuppression within the TME, leading to the aberrant expression of immune checkpoint markers such as PD-L1 on tumor cells; the subsequent binding of PD-L1 to the PD-1 receptor on T-cells effectively ‘switches off’ the anti-tumor cytotoxic T-cell response [10]. Furthermore, the presence of the TERT mutation is linked to a detrimental remodeling of the immune infiltrate, characterized by a reduction in cytotoxic CD8+ T lymphocytes and a simultaneous increase in immunosuppressive cell populations, including Myeloid-Derived Suppressor Cells (MDSCs) and Tumor-Associated Macrophages (TAMs), particularly the M2 phenotype, all of which actively suppress the immune system and promote cancer progression.

### 2.3. DAXX Mutations: Marker of High-Risk Disease

The DAXX gene (Death-domain Associated protein) encodes a protein that is crucial for several cellular functions, including transcription regulation, initiation of apoptosis (Programmed Cell Death) and telomere maintenance by the alternative lengthening of telomeres (ALT) pathway. Mutations in DAXX are generally loss-of-function mutations that lead to the inactivation or loss of the protein, which promotes tumorigenesis. They are associated with ATC and with PTC or FTC progression into a high-grade aggressive tumor. They tend to co-occur with a major driver mutation like BRAF or RAS [11]. The presence of a telomere elongation-associated mutation such as DAXX, often alongside ATRX [12] or TERT mutations, is a marker of high-risk disease primarily associated with the poorly differentiated and highly aggressive ATC, though they can also be found in other forms, typically co-occurring with other major driver mutations.

### 2.4. EIF1AX Mutations in Advanced Thyroid Cancer

The gene EIF1AX (Eukaryotic Translation Initiation Factor 1A X-linked) plays a critical role in the translation initiation complex, acting as a key regulator of the rate of protein synthesis. Mutations in *EIF1AX* are frequently observed in advanced thyroid cancers, particularly PDTC and ATC, and less commonly in PTC. These mutations tend to cluster in the protein’s termini, such as the A113 splice variant at the C-terminal tail or other variants in the N-terminal tail. A significant observation in aggressive thyroid cancers is the frequent co-occurrence of *EIF1AX* mutations with *RAS* mutations [13]. This co-occurrence suggests a powerful synergistic effect that substantially accelerates both tumorigenesis and disease progression. The core mechanism of *EIF1AX* mutations is a gain-of-function that results in an overall increase in the rate of protein synthesis and cell growth. The connection between mutant EIF1AX and the PI3K/AKT/mTOR pathway is fundamentally tied to up-regulation of protein synthesis that accelerates tumor growth, driving disease progression.

### 2.5. Aberrantly Activated Transduction Signaling Pathway

The PI3K/AKT/mTOR pathway (Phosphatidylinositol 3-kinase/Protein Kinase B/mammalian Target of Rapamycin) is a crucial signaling cascade that is frequently activated in various types of thyroid cancer, particularly in the more aggressive forms like FTC, PDTC, and ATC [14,15]. This pathway is a central regulator of many cellular processes vital for cancer progression, such as Cell Proliferation and Growth, inhibition of apoptosis, angiogenesis and general protein synthesis. The pathway is normally activated by growth factor receptors (Receptor Tyrosine Kinases or RTKs) on the cell surface (Table 2). Constitutive activation of this pathway drives the aggressive and de-differentiated phenotypes often seen in advanced thyroid cancers. The pathway’s activation is a strong indicator of a worse prognosis, especially in PTC patients who also have the BRAF-V600E mutation [16]. Mutation in the p110 catalytic subunit [17,18], the PI3K enzyme is a common alteration, resulting in an increased and upregulated production of the crucial second messenger PIP3 (Phosphatidylinositol (3,4,5)-trisphosphate) that is essential for recruiting and activating the next major protein in the cascade, AKT. Similarly, mutations in AKT itself or in the RAS family of small G proteins can also lead to hyperactivation of the pathway. Conversely, the pathway can be constitutively activated through the loss of function of its negative regulators. The most notable example is the tumor suppressor gene PTEN (Phosphatase and Tensin Homolog) [19]. PTEN normally acts as a brake on the pathway by reversing the action of PI3K—it removes a phosphate group from PIP3, converting it back into Phosphatidylinositol (4,5)-trisphosphate (PIP2) and thus turning off the signal. When the PTEN gene undergoes Loss-of-Function Mutations or has decreased expression, this crucial brake is removed. The accumulation of PIP3 goes unchecked, resulting in chronic, high-level activation of AKT and downstream signaling. Extracellular Matrix Protein 1 (ECM1) and Insulin-like Growth Factor Binding Protein 3 (IGFBP3) are known to regulate metastasis and epithelial-mesenchymal transition (EMT) through the PI3K/AKT pathway. Their overexpression in papillary cancer is related to a worse prognosis. Although p53 (Tumor Protein p53, encoded by the TP53 gene) is not a direct component of the pathway like PI3K, PTEN or AKT, is a highly relevant upstream regulator of the PI3K/AKT/mTOR pathway. P53 loss-of-function is a key event in the progression of more aggressive thyroid cancers. Its loss leads to sustained cell proliferation, survival, and metabolic changes, often marking a transition to more aggressive disease [20,21].

### 2.6. The RET Proto-Oncogene

The RET gene (REarranged during Transfection), located on chromosome 10, codes for a Receptor Tyrosine Kinase (RTK) protein. This protein sits on the cell surface and is normally involved in cell growth, survival, and differentiation. An activating mutation causes the protein to be constitutively active, driving uncontrolled C-cell proliferation and tumor formation. The most common mutations in familial MTC are point mutations affecting one of the five cysteine residues in the extracellular domain (Exons 10 and 11). These mutations cause the RET receptors to activate their internal tyrosine kinase domain without needing a growth factor signal [22] and correlate with the clinical presentation and aggressiveness of the disease. In sporadic MTC, somatic mutations regard RET mutations (M918T in codon 918) or non-Ret mutations that include RAS Family Mutations (HRAS, KRAS, or NRAS). Other pathways that can be altered in medullary carcinoma include the CTNNB1, RB1, and mTOR pathways.

### 2.7. The CTNNB1 Gene and β-Catenin

CTNNB1 [23] codes for β-catenin, a protein with a dual function according to its localizations: it is critical for cell–cell adhesion (forming part of adherens junctions) and is the central effector of Wnt signaling pathway. When the Wnt pathway is activated, beta-catenin accumulates in the cytoplasm, and then trans-locates to the nucleus, where it acts as a transcription co-factor, binding to transcription factors (TCF/LEF) that drive the expression of genes involved in cell proliferation and survival. CTNNB1 mutations that typically occur in Exon 3, prevent β-catenin from being phosphorylated and degraded, leading to its stabilization and constant nuclear accumulation, which promotes tumorigenesis. Prevalence of direct CTNNB1 mutations is rare in MTC although the Wnt-β-catenin pathway itself may be more relevant in MTC through cross-talk with the primary RET signaling pathway [24]. The RET oncogene, through its activation of the MAPK and PI3K/AKT pathways, is known to stimulate other growth and survival pathways as RET activation can trigger the β-catenin pathway indirectly. This suggests that while CTNNB1 itself may not be mutated in MTC, the β-catenin protein could still be activated and accumulated in the nucleus.

### 2.8. The Retinoblastoma (RB1) Pathway

Rb is a critical onco-suppressor gene that has been implicated in the development and progression of MTC, particularly in aggressive cases. While MTC is primarily driven by the RET proto-oncogene, the RB1 pathway provides a mechanism for uncontrolled cell proliferation, acting as a “second hit” or an important cooperative factor in tumorigenesis. The RB1 pathway governs the cell’s transition from the G1 phase (cell growth) to the S phase (DNA synthesis), effectively controlling the cell cycle checkpoint. RB1 gene encodes the Retinoblastoma protein (pRb), a tumor suppressor that is active (hypo-phosphorylated) in normal, resting cells, by binding to E2F transcription factors, thereby blocking the transcription of genes necessary for cell cycle progression (e.g., genes for DNA replication) [25]. When a cell receives a growth signal (like one from an activated RET receptor), pRb gets hyper-phosphorylated by the cyclin-dependent-kinases (CDK4/6 complex) that consequently release the E2F transcription factors, allowing them to move to the nucleus and activate the genes required for the cell to enter the S phase and divide. The RB1 pathway is functionally inactivated in MTC through mutations or losses in its regulatory components, leading to uncontrolled proliferation. In many sporadic MTC cases, especially those with aggressive features, studies have highlighted the frequent loss or mutation of genes that control phosphorylation of Rb protein, such as CDKN2C tumor suppressor gene. Deletions of the chromosomal region containing CDKN2C have been observed in sporadic MTC tumors and are associated with a worse prognosis, including the presence of distant metastasis at the time of diagnosis and decreased overall survival. The inactivation of the RB1 pathway, particularly through CDKN2C loss, often works in conjunction with the primary RET oncogene function as this promotes progression of the cell cycle by enhancing cyclin D expression, resulting in highly aggressive, uncontrolled proliferation.

### 2.9. MicroRNAs (miRNAs): Alterations of Transduction Pathways

A crucial role is also played by microRNAs (miRNAs)—small, non-coding RNAs that regulate gene expression and maintain cellular homeostasis. Their dysregulation contributes significantly to tumor progression and metastasis formation by acting as either oncomiRs (oncogenes) or tumor suppressors [26]. The most studied miRs are reported in Table 3. Their analysis not only provides prognostic information but also represents a potential target for new, tailored therapeutic strategies. The high expression of oncomiRs like miR-146b [27], miR-221, and miR-222 [28] is consistently reported in PTC and correlates with a worse prognosis, recurrence, and metastasis. Circulating levels of these miRNAs in serum or plasma are being investigated for non-invasive testing. Tumor suppressor miRNAs like miR-451a are downregulated in aggressive PTC [29]. Restoring the expression of these miRNAs in cancer cells [30,31,32] or inhibiting the function of the overexpressed oncomiRs (like miR-21 or miR-146b) using anti-miRs, represents a potential area for miRNA-targeted therapy to limit tumor progression and metastasis.

## 3. Papillary Thyroid Carcinoma: Etiology and Genetic Signature

PTC is the most prevalent form of thyroid malignancy, accounting for approximately 80% of all thyroid cancer cases [33,34,35]. It is typically characterized by a slow progression and an overall favorable prognosis. However, advanced and metastatic forms continue to pose significant challenges in clinical management. Over the past few decades, the global incidence of PTC has been rising—a phenomenon attributed not only to improved diagnostic techniques but also to increased exposure to genetic, environmental, and lifestyle factors.

From a molecular perspective, PTC is primarily driven by genetic alterations that activate the MAPK/ERK signaling pathway, leading to aberrant cell proliferation [36,37,38]. The most common mutations include RAS mutations, BRAF-V600E, and rearrangements of tyrosine kinase genes such as RET and NTRK [39,40,41,42]. These events result in distinct histological and biological phenotypes. The RAS-like tumors often maintain a predominantly follicular growth pattern and follow a less severe clinical course, and the BRAF-like tumors typically exhibit a more aggressive behavior, characterized by higher invasiveness and frequent lymph node involvement. A further critical group of alterations involves the TERT promoter, whose mutation is frequently associated with an unfavorable prognosis and adds a critical, highly aggressive layer of risk, particularly when combined with BRAF alterations [43]. TERT promoter mutations can be induced by exposure to ionizing radiation during childhood, one of the most significant risk factors for PTC development. Other conditions also contribute to tumor susceptibility, including elevated TSH levels, Hashimoto’s thyroiditis, iodine imbalances (both deficiency and excess), obesity, and exposure to industrial chemicals [44]. The tumor microenvironment (TME) plays a key role in PTC progression. Specifically, cancer-associated fibroblasts (CAFs) and extracellular matrix proteins—such as periostin—promote neoplastic growth by activating the integrin–FAK–STAT3 signaling pathways [45,46,47]. Further regulation is exerted by microRNAs (miRNAs), whose dysregulation drives proliferation, invasion, and metastasis [48].

### 3.1. RAS-like PTC (RL-PTC)

This group is driven by point mutations in the RAS family genes (N-RAS, H-RAS, K-RAS), which are found in about 10–20% of PTCs [49,50] or by rearrangements like PAX8/PPARγ [51]. The RAS family mutations represent a class of alterations that, like the BRAF mutation, lead to the constitutive activation of the MAPK signaling pathway, but generally with a lower oncogenic potential than BRAF V600E. The Paired Box 8/Peroxisome Proliferator-Activated Receptor Gamma (PAX8/PPARγ) rearrangement is a chromosomal translocation that fuses the PAX8 gene (a thyroid-specific transcription factor) with the PPARγ gene (a nuclear receptor), also contributing to tumorigenesis, often in tumors with a follicular growth pattern [52]. Tumors driven by RAS mutations or PAX8/PPARγ rearrangements are typically associated with a less aggressive, indolent, or low-risk phenotype when compared to those with the BRAF V600E mutation. They are strongly linked to a specific histology, primarily the Follicular Variant of PTC (FVPTC), especially the encapsulated form, and are the characteristic drivers of Non-Invasive Follicular Thyroid Neoplasm with Papillary-like Nuclear Features (NIFTP), which has a near-benign behavior [53,54]. In terms of invasiveness, these genetic changes are associated with a lower incidence of Extrathyroidal Extension (ETE) and Lymph Node Metastasis (LNM) at diagnosis. Consequently, they are generally associated with a more favorable prognosis and excellent long-term survival. Crucially for treatment, this group of tumors tends to maintain a higher degree of thyroid differentiation. This is a significant advantage, as the cancer cells often retain the ability to concentrate iodine, leading to better Radioiodine (I^131^) uptake and a more effective response to I^131^ therapy for advanced or recurrent disease compared to the less-differentiated, BRAF-driven tumors. Therapeutic options for PTC are now broad and personalized based on specific clinical and biological factors, such as the patient’s age, tumor size and location, presence of metastases, and the tumor’s genetic-molecular profile. The multi-modal therapeutic approach aims to ensure optimal disease control while minimizing morbidity and the risk of recurrence.

### 3.2. BRAF-like PTC (BL-PTC)

This subtype is named for the common driver mutation, BRAF-V600, which is the most frequent genetic alteration in PTC (found in approximately 40–50% of cases) and is a primary driver because it causes the constitutive activation of the MAPK (Mitogen-Activated Protein Kinase) signaling pathway [55]. This pathway regulates cell proliferation, differentiation, and survival, and its constant ‘on’ state leads to uncontrolled cell growth. While the BRAF-V600E mutation is the main driver, other genomic alterations like certain gene fusions (e.g., RET/PTC) can also contribute to papillary cancer [56]. The presence of the BRAF-V600E mutation is generally associated with a more aggressive, high-risk phenotype of thyroid cancer. It is specifically linked to more aggressive histological variants of PTC, such as the Tall Cell Variant, Hobnail Variant and the SVPTC [57].

Regarding invasiveness, tumors with this mutation show significantly higher rates of Extrathyroidal Extension (ETE) and Lymph Node Metastasis (LNM) upon initial diagnosis, indicating a greater propensity for local spread. As a result, the prognosis for patients whose tumors harbor the BRAF-V600E mutation is typically poorer. They face an increased risk of disease recurrence and a reduced rate of overall survival compared to patients with other mutations or wild-type BRAF. The mutation can also impact treatment response. Tumors with BRAF-V600E often exhibit lower radioiodine avidity (less differentiation). This poor uptake of I^131^ can potentially complicate the effectiveness of Radioiodine (I^131^) therapy when used to treat advanced or recurrent disease [58,59,60,61].

## 4. Follicular Thyroid Carcinoma: Clinical and Molecular Insights

FTC is the second most common malignancy originating from thyroid follicular cells, accounting for approximately 6–15% of differentiated thyroid tumors [62]. Its prevalence is inversely related to iodine intake: in iodine-sufficient areas, it accounts for about 10% of malignant thyroid neoplasms, but in iodine-deficient regions, this figure can reach 25% to 40%. Although generally slow-growing, FTC is considered more aggressive than PTC. It exhibits a pronounced tendency for hematogenous spread and presents with heterogeneous clinical behavior.

### 4.1. Histopathological Characteristics and Invasive Variants

From a histopathological perspective, FTC is characterized by uniform follicular growth, lacking the typical nuclear changes seen in PTC. It is classified into minimally invasive, encapsulated angioinvasive, and widely invasive forms. Vascular invasion is the critical determinant of aggressiveness and prognosis: when it exceeds 2–4 foci, it is associated with a significantly increased risk of metastasis and mortality. Encapsulated variants with marked angioinvasion can be clinically more aggressive than widely invasive forms lacking vascular invasion. Distant metastases are the main adverse feature of FTC, with a prevalence reaching 45% in widely invasive forms, and can, albeit more rarely, also occur in minimally invasive forms. The most common sites are the lungs, bones, liver, brain, and skin. Metastatic incidence is higher in elderly patients, while lymph node involvement is uncommon, observed in less than 10% of cases [63].

### 4.2. Molecular Profile in FTC

Molecular analysis has revealed a unique genetic profile that distinguishes FTC from PTC. RAS family mutations (NRAS, HRAS, KRAS) are the most frequent alterations (30–50%), typically located at codon 61 of NRAS/HRAS and codons 12/13 of KRAS. PAX8-PPARγ rearrangements, present in 10–40% of cases, are associated with a younger age at diagnosis and often with more invasive forms, even in the presence of small tumors. TERT promoter mutations are of particular prognostic significance, correlating with increased aggressiveness, reduced response to radioiodine, and a worse prognosis, especially when coexisting with RAS mutations [64]. The PAX8/PPARγ rearrangement also known as PAX8-PPARγ fusion, is a significant genetic alteration frequently found in Follicular Thyroid Carcinoma (FTC) and some follicular-patterned lesions, such as the non-invasive follicular thyroid neoplasm with papillary-like nuclear features (NIFTP). This genetic alteration occurs when a piece of chromosome 2, containing the PPARγ gene, breaks off and fuses with a piece of chromosome 3, containing the PAX8 gene. This is a transcription factor for thyroid-specific genes (like thyroglobulin). PPARγ is a nuclear receptor that plays a role in lipid metabolism and cell differentiation. It often acts as a tumor suppressor. The resulting fusion gene, PAX8/PPARγ consists of the active promoter and a portion of the PAX8 gene fused to the entire coding region of the PPARγ gene, whose protein drives the growth and development of the tumor primarily with two different mechanisms. The first one is a dominant-negative effect as it binds to the DNA sites normally targeted by the wild-type protein, but it fails to activate the proper differentiation pathways, inhibiting the tumor-suppressive function of normal PPARγ protein. The second mechanism is due to the over-expression of the fusion protein for PAX 8 active promoter activity that functions as an abnormal transcription factor, potentially regulating the expression of genes involved in cell proliferation, thus promoting cancerous growth [65].

### 4.3. Clinical and Biological Prognostic Factors

Poorer clinical outcomes are associated with an age over 45–55 years, tumor size greater than 4 cm, the presence of at least two foci of vascular invasion, a Ki-67 [66] proliferative index ≥ 4–5%, rapid thyroglobulin doubling time, and a neutrophil/lymphocyte ratio > 3 in refractory metastatic forms. These findings have encouraged a progressive shift towards personalized therapeutic strategies, particularly for patients with extensive or large tumors or distant metastases [67].

## 5. Anaplastic Thyroid Carcinoma

ATC is a rare but unequivocally the most aggressive and lethal form among thyroid cancers. Although it constitutes a small percentage of thyroid carcinoma diagnoses, it is responsible for the majority of thyroid cancer-related deaths [68]. Patients with ATC face a rapidly progressive course, with a median survival of only 5 months and a one-year survival rate hovering around 20% [69]. ATC predominantly manifests between the fifth and seventh decades of life and, similar to other thyroid tumor forms, is more common in females (approximately 60% of cases) [70]. Known risk factors include long-standing goiter (observed in up to 80% of patients), blood group B, obesity, and, crucially, malignant transformation from a pre-existing DTC. While it remains controversial whether ATC can arise de novo, the frequent finding of residual areas of DTC or PDTC within the tumor mass supports the prevailing hypothesis of evolutionary de-differentiation.

### 5.1. Clinical and Morphological Aspects

Clinically, ATC presents as a rapidly and relentlessly growing cervical mass, often associated with acute compressive symptoms such as dysphagia, dyspnea, hoarseness, and local pain. At the time of diagnosis, invasion of vital locoregional structures, including the trachea, esophagus, recurrent laryngeal nerve, and lymph nodes, is common [71]. Furthermore, many patients exhibit synchronous or impending metastasis involving the lungs, bones, liver, and brain. Morphologically, ATC is characterized by extreme heterogeneity, displaying pleomorphic cells with large nuclei, multinucleated giant cells, and spindle cells or squamoid populations [72]. The lack of thyroid differentiation features is its hallmark. Irrespective of the predominant pattern, all these tumors exhibit a Ki-67 proliferative index typically greater than 30%, high rates of atypical mitoses, and extensive necrosis, all indicators of high genomic instability and aggressiveness. As there is no prognostic correlation among the morphological subtypes, ATC is universally classified as undifferentiated high-grade carcinoma and is always assigned to Stage IV upon diagnosis, subdivided into [73]:

- Stage IVA: Neoplasm confined to the thyroid without nodal or distant metastases.
- Stage IVB: Locoregional invasion and/or lymph node metastasis.
- Stage IVC: Distant metastasis.

### 5.2. Origin and De-Differentiation Process

The pathogenesis of ATC is viewed as a dynamic and multiphase phenomenon of progressive loss of differentiation. The hypothesis of evolutionary dedifferentiation is supported by the frequent clinical history of pre-existing thyroid carcinoma, found in 58–90% of patients, and the presence of foci of DTC or PDTC within the tumor. The transformation is not a single event but the consequence of a progressive series of molecular alterations (activating or inactivating mutations) that accumulate. These alterations disrupt the cell cycle and signal transduction pathways, culminating in uncontrolled proliferation, genomic instability, and the gradual shift from the differentiated phenotype to PDTC and finally to the anaplastic phenotype. However, evidence of significant molecular divergence in some mixed DTC/ATC tumors suggests that a de novo onset cannot be entirely ruled out [74].

*Genomic Instability in ATC*

Molecular analysis is essential for understanding pathogenesis and guiding therapy, distinguishing between early mutations and late mutations [75]. Early mutations, such as BRAF and RAS, are common in differentiated carcinomas and can persist in derived ATCs. The V600E mutation is observed in a significant percentage (40–70%) of ATCs, particularly those with a coexisting PTC component, indicating an initiating event. Mutations in the RAS family genes (NRAS, HRAS, KRAS) are found in 15–40% of cases. The most distinctive and frequent alteration is the mutation (50–70% of cases), leading to the loss of p53 tumor suppressor function. Although not specific to ATC, abnormal p53 immunoreactivity supports the diagnosis. Other key mutations include those in the TERT promoter (65–75% of cases) and alterations in the PI3K/AKT pathway, which are also late events and often coexist with early mutations [76]. Beyond the common driver mutations (BRAF, RAS, TP53), the accumulation of epigenetic changes and the presence of rare but therapeutically relevant gene fusions like striatin/anaplastic lymphoma kinase (STRN-ALK) are critical for tumor progression and treatment planning. Epigenetic alterations involve changes in gene expression without altering the underlying DNA sequence. These changes contribute significantly to the dedifferentiation process that defines ATC by silencing tumor suppressor genes and activating oncogenic pathways. Key complexes involved in ATC include chromatin remodeling and histone modification machinery. The Switch/Sucrose Non-Fermentable complex (SWI/SNF) is a large, multi-subunit ATPase-dependent chromatin remodeler. It plays a fundamental role in controlling gene expression by physically altering the structure of chromatin, making genes accessible or inaccessible for transcription. In ATC, mutations or loss of function in components of the SWI/SNF complex, such as ARID1A, SMARCA4, and PBRM1, are frequently observed [77]. Loss of function in these tumor suppressor genes leads to aberrant chromatin accessibility, resulting in the silencing of genes necessary for thyroid differentiation (like those involved in iodine uptake) and the activation of pathways that promote aggressive growth and the mesenchymal phenotype characteristic of ATC. Histone methyltransferases (HMTs) regulate gene expression by adding methyl groups to specific lysine or arginine residues on histone tails. This modification can either activate or repress transcription [78]. Alterations in HMTs are often implicated in ATC. For example, mutations or loss of function in the SETD2 gene, which encodes a histone methyltransferase responsible for H3K36me3 (a mark associated with active transcription), have been reported [79]. The resulting disruption of normal histone modification patterns leads to mis-regulation of the transcriptome, driving uncontrolled proliferation and promoting genomic instability—a hallmark of high-grade malignancy. These changes collaborate with genetic driver mutations to push the tumor toward a fully anaplastic phenotype. Gene fusions occur when two previously separate genes become physically joined, resulting in a novel chimeric protein that often exhibits constitutive oncogenic activity. These are particularly important because they are frequently actionable with targeted therapies. The fusion STRN-ALK is a rare but highly significant event observed in a subset of ATC [80]. The ALK gene is a tyrosine kinase receptor (RTK). In the fusion protein, the N-terminal part of STRN provides a dimerization domain, which forces the fusion protein to form stable dimers. This dimerization leads to the constitutive activation of the C-terminal ALK kinase domain, irrespective of external signaling. Constitutively active ALK drives pro-survival and proliferative signaling cascades, including the MAPK and PI3K/AKT pathways, contributing to the aggressive behavior of ATC.

### 5.3. Extracellular Vesicles (EVs) from Thyroid Tumor Cells

The field of extracellular vesicles (EVs)—which include microvesicles (or ectosomes) and exosomes—is one of the most rapidly advancing areas in thyroid cancer research [81]. These nanometer-sized, lipid bilayer-enclosed particles are released by nearly all cells, including thyroid tumor cells, and serve as crucial mediators of intercellular communication in both physiological and pathological processes. Thyroid cancer-derived EVs are now recognized as active shuttles that transfer complex biological information to surrounding and distant cells, promoting an aggressive tumor phenotype. EVs are heavily implicated in driving EMT, a key process where epithelial cells acquire a migratory, invasive, fibroblast-like phenotype, which is the first step toward metastasis. Studies show that EVs from ATC and PTC cells can transfer specific molecular cargo (such as miRNAs like miR-221/222) that alters the recipient cell’s phenotype, contributing to the acquisition of malignancy and invasion [82]. EVs released by tumor cells and surrounding stromal cells (like fibroblasts) engage in crosstalk that creates a pro-tumorigenic milieu. Tumor-derived EVs, particularly under stressful conditions like hypoxia, can induce the secretion of factors like proMMP2 from normal fibroblasts, promoting a favorable environment for invasion and metastasis [83]. Exosomes from PTC cells, especially those subjected to hypoxia, have been shown to enhance the growth of new blood vessels (angiogenesis) by carrying specific miRNAs (e.g., miR-21-5p). This cargo can directly target and suppress genes like *TGFBI* and *COL4A1*, leading to increased endothelial tube formation and facilitating tumor spread. The biological activity of EVs is determined by their cargo, which reflects the molecular signature of the originating tumor cell. EVs carry various forms of genetic material, including mRNAs, microRNAs (miRNAs), long non-coding RNAs (lncRNAs), and even circRNAs and fragments of DNA. Specific miRNAs, such as miR-146b-5p and miR-222-3p, found in PTC-derived exosomes are associated with promoting migration, invasion, and lymph node metastasis [84,85]. Tumor-derived EVs contain proteins involved in promoting cancer hallmarks. For instance, the stress/danger-associated molecule HMGB1 (High-Mobility Group Box 1) is found in thyroid cancer EVs, where it can promote tumor-related inflammation and progression by activating signaling pathways like NF-kB [86,87]. The high stability of EVs in bodily fluids and their representation of the tumor’s molecular status make them a promising tool for liquid biopsy. As EVs can be isolated from biological fluids (e.g., blood, urine, saliva), the analysis of their specific molecular cargo (proteins, miRNAs) is being actively investigated to differentiate between benign and malignant thyroid nodules, potentially reducing the need for repeated or unnecessary fine-needle aspiration biopsies (FNABs) or to predict local metastasis (e.g., high expression of exosomal miR-485-3p correlates with extrathyroidal extension and lymph node metastasis in PTC) [88]. As well as their diagnostic and prognostic use, research is exploring the use of engineered EVs as natural drug carriers for targeted therapy, offering an approach with stable membrane structure and better biocompatibility to counteract drug resistance in advanced thyroid cancer [89].

### 5.4. Tumor Microenvironment (TME) in ATC

ATC is characterized by a highly complex and immunosuppressive TME, dominated by a massive infiltration of Tumor-Associated Macrophages (TAMs), which can account for 40% to 70% of the tumor mass. These TAMs, primarily of the pro-tumorigenic M2 phenotype, play a crucial role in the immunosuppressive tumor stroma, contributing to treatment resistance and the unfavorable prognosis of ATC [90,91]. Transcriptomic studies have identified a 78-gene signature associated with M2-TAMs capable of distinguishing ATC from Differentiated Thyroid Carcinomas (DTCs) that promote dedifferentiation, proliferation, migration, and invasion through the activation of the Wnt-beta-catenin and PI3K/AKT/mTOR pathways. This activation is mediated by the secretion of Wnt1/Wnt3a and Insulin-like Growth Factor (IGF), respectively. Another feature of M2 infiltrating macrophages is the expression of CD47, a protein that typically inhibits macrophage-mediated phagocytosis [92]. Preclinical studies have shown that the use of anti-CD47 antibodies can induce the phagocytosis of tumor cells by macrophages, suggesting a possible therapeutic approach to overcome the TAM-driven immune evasion. The immune microenvironment of ATC is often heterogeneous: approximately 50% of cases show an immune-depleted environment, while 34% display a “hot” microenvironment with high expression of inhibitory immune checkpoints, including CTLA-4, PD-L1, PD-L2, and TIGIT [93]. These checkpoints suppress the activity of cytotoxic T cells and promote the differentiation of T regulatory cells. Immunotherapy with ICIs, such as anti-PD-1, anti-PD-L1, and anti-CTLA-4 antibodies, has opened new therapeutic avenues for ATC [94]. The high prevalence of TAMs and the complex regulation of immune checkpoints underscore the importance of systematic screening for PD-L1status and, potentially, MMR markers (Mismatch Repair deficiency) to select patients most likely to benefit from immunotherapy.

## 6. Oncocytic Thyroid Carcinoma: Pathobiology, Genetics, and Management

OTC, also known as Hürthle Cell Carcinoma, is a malignant neoplasm derived from thyroid follicular cells, accounting for approximately 5% of all thyroid cancers. In 2022, the World Health Organization (WHO) recognized it as a distinct entity, separating it from Follicular Thyroid Carcinoma based on unique histopathological and molecular characteristics.

### 6.1. Epidemiology and Pathogenesis

OTC is diagnosed more frequently in women (female-to-male ratio 1.6–4.8:1) and typically presents between the ages of 50 and 60. While it can exhibit aggressive behavior, the overall 5-year relative survival remains high (approximately 98.5% across all stages). Predisposing factors include pre-existing thyroid pathologies (adenomas, goiter) and genetic syndromes such as Multiple Endocrine Neoplasia type 1 (MEN1) and familial non-medullary thyroid carcinoma. The tumor originates from oncocytic cells, a specialized subtype of follicular cells defined by a high density. These cells are characterized by voluminous nuclei with central nucleoli, loss of cell polarity, and granular eosinophilic cytoplasm rich in mitochondria.

### 6.2. Histopathology and Prognostic Subtypes

Oncocytic carcinoma is distinguished from oncocytic adenomas by a thick capsule, a solid or trabecular growth pattern, a high mitotic count, and frequent vascular or extrathyroidal invasion. OTC shows a higher metastatic risk compared to other differentiated forms, with both lymph node and distant spread, particularly to the lungs and bones (up to 13% of cases). Angiogenesis within the tumor microenvironment plays a crucial role in progression [95].

The biological behavior depends on the extent of capsular and vascular invasion, leading to three main prognostic subtypes:

- Minimally Invasive: Invasion confined exclusively to the capsule.
- Encapsulated Angioinvasive: Vascular invasion is limited (l–4 foci) or extensive (>4 foci).
- Widely Invasive: Capsular rupture and/or extension into the surrounding thyroid parenchyma.

Retrospective studies have demonstrated that OTC with extensive vascular invasion carries a significantly higher risk of recurrence compared to follicular carcinoma with similar features.

### 6.3. Mitochondrial and Genomic Instability in OTC

**Figure 1:**
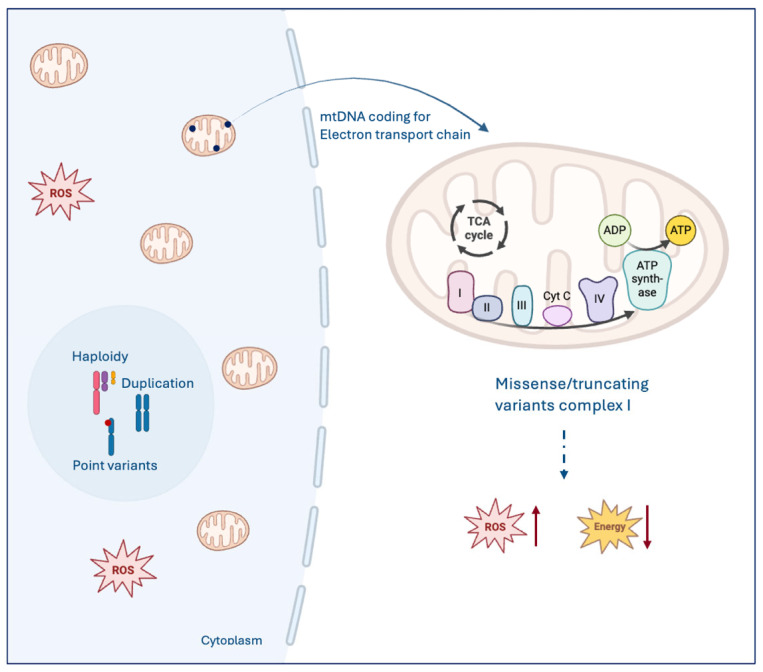
Core mechanism underlying the oncocytic phenotype and molecular pathology of Oncocytic Carcinoma (OC). Pathogenic somatic mutations and deletions in mitochondrial DNA (mtDNA) lead to a partial or complete loss of Complex I function within the Electron Transport Chain (ETC) that generate increased levels of Reactive Oxygen Species (ROS) and drop in energy levels.

The most striking cytological feature of OTC cells—their abundant, granular, eosinophilic cytoplasm (the oncocytic phenotype) is a direct consequence of massive mitochondrial accumulation. This hyper-proliferation, however, is paradoxical as the mitochondria are often defective due to somatic mutations in their own genome, the mtDNA. A hallmark of OTC is the high prevalence of pathogenic mutations and large-scale deletions in mtDNA, with a strong enrichment in genes encoding subunits of Respiratory Complex I (NADH-ubiquinone oxidoreductase) of the electron transport chain (ETC) [96]. Genes such as *ND1*, *ND2*, *ND4*, and *ND5* [97] are frequently affected. These disruptive mutations lead to a partial or complete loss of Complex I function, resulting in severely reduced efficiency of oxidative phosphorylation (OXPHOS) and a subsequent drop in ATP synthesis driven by mitochondrial respiration. Cells attempt to overcome this bioenergetic deficiency through a process known as the compensatory theory or mitochondrial biogenesis. This involves the proliferation of mitochondria, resulting in the characteristic oncocytic appearance [98]. The dysfunctional ETC, specifically at Complex I and potentially Complex III, also leads to an increased production of Reactive Oxygen Species (ROS) (Figure 1). This ROS hyper-production can inflict further damage on the highly susceptible mtDNA, creating a vicious cycle that perpetuates mitochondrial defects and drives further instability In addition to mitochondrial changes, malignant OTC is defined by a high degree of Genomic Loss of Heterozygosity (gLOH) and Chromosomal Instability (CIN), a feature that clearly distinguishes it from typical FTC and is linked to poor outcomes.

The genomic instability presents as a spectrum, with minimally invasive tumors often displaying a near-diploid genome with only a few chromosomal alterations. However, as the tumor evolves toward aggressive, widely invasive forms, it progresses toward marked aneuploidy, frequently involving genome haploidization (monosomies of entire chromosomes) followed by endoreduplication, resulting in a near-diploid or polyploid state with widespread chromosomal losses. This massive gLOH affects large portions of the genome, indicating a severe defect in cell division and DNA repair mechanisms. A particularly significant and recurrent finding in aggressive OTC is the frequent duplication of Chromosome 7. Importantly, while the majority of chromosomes exhibit extensive gLOH, Chromosome 7 often escapes this loss and is retained in a heterozygous state, often followed by a full duplication, leading to a retained or increased copy number (trisomy or tetrasomy) [99]. This specific retention and duplication suggest that maintaining or increasing the dosage of key oncogenes located on Chromosome 7 is crucial for tumor survival and progression. The key genes implicated include EGFR (Epidermal Growth Factor Receptor), BRAF (though typically non-V600E), and MET (Mesenchymal–Epithelial Transition factor). The duplication of Chromosome 7, especially when associated with the process of genome haploidization and endoreduplication, is therefore considered a strong molecular marker for aggressive behavior and is associated with an unfavorable prognosis in OTC patients. The distinct combination of mitochondrial defects and this unique pattern of genomic instability, especially the retention of Chromosome 7, defines the molecular landscape and clinical aggressiveness of OTC.

### 6.4. Immunosuppressive TME in OTC

OTC tumors, particularly the more aggressive widely invasive phenotype, often exhibit a strikingly immune-depleted microenvironment [100]. This low level of immune cell infiltration is a key finding that differentiates OTC from other thyroid cancer subtypes, which may be more immune-infiltrated (or “hot”). There is a negative correlation between gLOH and the level of immune infiltration as tumors exhibiting profound genomic instability have the lowest level of immune infiltration and are associated with a greater likelihood of recurrence. This is probably due to a reduction in the expression of crucial antigen-processing and presentation machinery (APM) components on the tumor cell surface, which are necessary for immune recognition. Furthermore, the extensive gLOH may reduce the neoantigen burden or alter the expression of molecules that recruit immune cells, effectively cloaking the tumor from cytotoxic T cell responses. Furthermore, despite the overall “cold” or immune-depleted nature of the OTC TME, certain immunosuppressive elements are notably present, suggesting active immune evasion mechanisms. Marked upregulation of Programmed Death-Ligand 1 (PD-L1) is frequently observed in widely invasive OTC). PD-L1 is an immune checkpoint molecule that binds to the PD-1 receptor on T cells, effectively deactivating them. Its heightened expression suggests that even the few infiltrating T cells are being functionally suppressed, making immune checkpoint inhibition a potential therapeutic strategy for these aggressive tumors. The TME in OTC like in other aggressive thyroid tumors is noted to have a significant presence of myeloid cells, including Tumor-Associated Macrophages (TAMs), which is associated with increased PD-L1 expression [101]. Macrophages, particularly the M2-polarized phenotype, are known to promote immune suppression, angiogenesis, and tissue remodeling, thereby fostering tumor growth and metastasis. As the tumor progresses and de-differentiates, there is a trend toward an increase in myeloid cells and fibroblasts in the TME, which mirrors the shift observed in other de-differentiated thyroid cancers.

### 6.5. Prognosis

Follow-up relies on clinical exams, neck ultrasound, and thyroglobulin monitoring for early recurrence identification. Prognosis depends on tumor size, extrathyroidal extension, age (55 years old), lymph node involvement, and vascular invasion. Small, confined tumors have an excellent prognosis (10-year survival rates = 90%), while advanced or metastatic disease significantly worsens survival.

## 7. Medullary Thyroid Cancer: Molecular Alterations and Advances in Diagnosis, Prognosis and Therapy

MTC arises from the parafollicular C cells (or C-cells) of the thyroid gland [102], which derive from the neural crest and release calcitonin. High levels of calcitonin and carcinoembryonic antigen (CEA) in the blood are the key diagnostic and follow-up markers for MTC. It is classified into a sporadic form (75% of cases) and a hereditary form (25% of cases) that associates to multiple endocrine neoplasia type 2 (MEN 2). The MEN 2 form includes MEN 2A, MEN 2B [103] and Familial MTC. The pathogenesis of MTC is fundamentally driven by activating mutations in the RET proto-oncogene. However, the transition toward a more aggressive, highly malignant phenotype is frequently precipitated by the suppression of the Rb tumor suppressor (described in Section 2).

### 7.1. Advances in MTC, Management and Prognosis

Recent advancements are significantly improving the diagnosis, risk stratification, and potential treatment of Medullary Thyroid Carcinoma (MTC). An International Grading System for MTC has recently been introduced [104]. This new system is designed to aid clinical decision-making by providing a more accurate prognostic prediction and better management strategies for sporadic MTC cases.

### 7.2. Genetic Testing and Prophylaxis

Genetic testing for germline *RET* mutations remains essential for all MTC patients to accurately differentiate between sporadic and hereditary disease (specifically, Multiple Endocrine Neoplasia type 2—MEN 2). The early identification of this mutation allows for prophylactic thyroidectomy in affected family members, which significantly increases the cure rate for hereditary cases [105].

## 8. Diagnosis of Thyroid Tumors

### 8.1. Initial Nodule Assessment: Imaging and General Techniques

Recent diagnostic advancements have transformed the clinical management of thyroid nodules. High-Resolution Ultrasonography (HRUS) is now the primary imaging technology for identifying and monitoring all thyroid nodules [106]. This is often supplemented by Fine Needle Aspiration Cytology (FNAC), elastography, which provides a better distinction between benign and malignant lesions by assessing tissue stiffness, thereby reducing the number of unnecessary invasive procedures [107,108]. Advanced techniques such as Computed Tomography (CT), Magnetic Resonance Imaging (MRI), and PET/CT provide additional information in more complex cases. Specifically, PET/CT is valuable for detecting metastases or recurrence, enhancing diagnostic accuracy in advanced forms of the disease [109].

### 8.2. Diagnosis and Molecular Markers of PTC

In parallel with clinical advances, molecular diagnostics have enabled the identification of genetic markers and gene expression profiles that refine the prognosis and risk stratification of PTC. Differential expression of genes like IGFBP3 and ECM1 helps distinguish metastatic from non-metastatic PTC [110]. Crucially, the detection of the BRAFV600E mutation remains one of the most significant achievements in PTC molecular diagnosis. This mutation is associated with a higher incidence of lymph node metastasis and increased mortality, establishing it as a fundamental indicator of tumor risk and biological aggressiveness.

### 8.3. Diagnosis and Management of FTC and OTC

Preoperative diagnosis of FTC and OTC remains a major challenge. Unlike PTC, fine-needle aspiration (FNA) cannot assess the definitive diagnostic criterion—capsular and/or vascular invasion—which distinguishes carcinoma from follicular adenoma or oncocytic adenoma. Consequently, the definitive diagnosis for nodules with indeterminate cytology (Bethesda III–IV) often relies on histopathological examination after lobectomy [111] and an estimated malignancy risk between 13% and 34%. Molecular testing is utilized for preoperative risk stratification in these indeterminate nodules. A “benign” result has a very high Negative Predictive Value (NPV), meaning it can reliably rule out cancer and may spare the patient diagnostic surgery. Conversely, the identification of common thyroid cancer mutations, such as BRAF or specific RET fusions, strongly suggests malignancy and guides the patient toward surgery. In FTC/OTC, specific molecular markers predict tumor behavior, influencing the extent of the initial surgery. The TERT promoter mutation correlates strongly with greater aggressiveness and reduced radioiodine response, favoring total thyroidectomy [112]. While RAS mutations (NRAS, HRAS, KRAS) are frequent in follicular-patterned lesions, their presence supports surgery, especially when coexisting with TERT mutations, indicating a higher-risk profile. PAX8-PPARγ rearrangements are associated with more invasive forms. By integrating these high-risk mutations and profiles with clinical and ultrasound findings, physicians achieve accurate risk stratification, enabling personalized therapy.

### 8.4. Diagnostic Approach in ATC

The ATC genome is defined by extremely high instability. The diagnostic approach for ATC must be rapid and multidisciplinary, given the potential for acute airway compromise. While Fine-Needle Aspiration Cytology (FNAC) is used, a core or excisional biopsy is often necessitated for complete histological and immunohistochemical (IHC) characterization. The differential diagnosis is limited but crucial, particularly against PDTC, primary thyroid lymphoma, thyroid squamous cell carcinoma, or metastases from other sites. IHC is fundamental for confirming the undifferentiated nature and thyroid origin of ATC. ATC typically shows loss of expression of thyroid differentiation proteins, such as thyroglobulin (TG) and thyroid transcription factor 1 (TTF-1), a sign of its undifferentiated nature. Significant expression of these markers would suggest a higher degree of differentiation and the plausibility of alternative diagnoses. The expression of PAX8, is retained in about 40–60% of cases, although often focally, supporting the ATC diagnosis in an appropriate morphological context. Pan-cytokeratins are useful for confirming epithelial nature, especially in spindle or giant cell variants. Ki-67, with an index typically greater than 30%, confirms aggressive proliferation. Routine IHC panels, which include cytokeratins, *TG*, *TTF-1*, *BRAF V600E*, *PAX8*, *Ki-67* [113] *p53* [114] and other markers, help distinguish ATC from other thyroid neoplasms or metastases. In tumors with a history of DTC, the well-differentiated areas show intact expression of *TG*, *TTF-1*, and *PAX8*, supporting the model of in situ transformation.

The use of Next-Generation Sequencing (NGS) is essential for screening ATC patients to identify these rare, actionable fusions, underscoring the shift toward precision oncology in this highly lethal disease.

### 8.5. Diagnostic Approach in MTC

The diagnostic approach for MTC is multifaceted, relying on a combination of cytopathology, biochemical markers, immunohistochemical confirmation, advanced genetic testing, and sophisticated molecular imaging to accurately identify, stage, and prognosticate the disease. This careful, integrated strategy is essential because MTC can sometimes be a great mimicker of other thyroid malignancies, complicating initial identification [115].

#### 8.5.1. Advanced Imaging and Staging

Anatomical imaging, primarily high-resolution neck ultrasound, is used initially to characterize the thyroid nodule and assess the status of the cervical lymph nodes, as MTC has a strong propensity for early lymphatic spread. However, for staging advanced, recurrent, or metastatic disease, functional and molecular imaging techniques offer superior sensitivity. Unlike differentiated thyroid cancers which are imaged using iodine, MTC requires specialized neuroendocrine tracers. Positron Emission Tomography-Computed Tomography (PET/CT) utilizing radiolabeled amino acids like F-fluoro-3,4-dihydroxyphenylalanine (F-FDOPA) or somatostatin analogs like 68-Ga-DOTATATE is particularly effective [116]. These advanced imaging techniques ensure that the full extent of the neuroendocrine malignancy is accurately mapped, allowing for precise surgical planning and systemic therapeutic intervention.

#### 8.5.2. Initial Cytological and Biochemical Diagnostics

The key biochemical hallmark of MTC is the production and secretion of calcitonin (CTN) into the blood [117]. Consequently, serum calcitonin is the most reliable circulating diagnostic marker and is routinely used for screening, initial diagnosis, and post-treatment monitoring. The primary step in evaluating a suspicious thyroid nodule is typically ultrasound-guided FNAC. While FNAC provides the initial cellular architecture, yielding cells that may present with a classic “salt-and-pepper” chromatin pattern characteristic of neuroendocrine tumors, it is often insufficient on its own for a definitive diagnosis due to the high variability in MTC cell morphology. To enhance the diagnostic specificity of the cytological sample calcitonin levels are measured on the same samples. Carcinoembryonic antigen (CEA) is another marker frequently co-secreted by C cells, and while less specific for MTC than calcitonin, its levels are also closely monitored. In fact, a rapid doubling time of CEA is often considered a more reliable indicator of poorer prognosis and aggressive disease behavior than calcitonin doubling time, guiding further clinical management.

#### 8.5.3. Histopathology and Immunohistochemical Confirmation

Once tissue material is available, either from the initial FNAC or post-surgical resection, the diagnosis is confirmed by histopathological analysis and the application of immunohistochemistry (IHC) [118]. The tumor cells are typically positive for neuroendocrine markers, prominently staining for Calcitonin and Chromogranin A, confirming their C-cell origin. The presence of stromal amyloid deposition, which is derived from the calcitonin protein, is a classic finding, although it is not universally present. The combination of characteristic cell morphology, such as plasmacytoid or spindle cells with the defining “salt-and-pepper” nuclei, and positive immunostaining for Calcitonin, is crucial for separating MTC from other thyroid lesions, such as papillary carcinoma variants which stain positively for Thyroglobulin and TTF-1 but negatively for Calcitonin. A critical component of the MTC diagnostic workflow is genetic testing due to the significant hereditary link. Approximately twenty-five percent of MTC cases are inherited, often presenting as part of the Multiple Endocrine Neoplasia type 2 (MEN 2) syndrome, which includes subtypes like MEN 2A and MEN 2B. Therefore, all patients diagnosed with MTC must undergo germline genetic analysis of the RET proto-oncogene. Identifying a germline RET mutation has profound implications, dictating the need for prophylactic surgery in asymptomatic family members and mandatory screening for associated tumors like pheochromocytoma and hyperparathyroidism. Beyond hereditary assessment, somatic RET mutations, such as the M918T mutation, are also investigated in sporadic MTC cases as they correlate with more aggressive disease and are pivotal for guiding personalized therapeutic strategies, specifically indicating suitability for RET-targeted tyrosine kinase inhibitor therapy.

### 8.6. The Use of Artificial Intelligence in Thyroid Cancer Diagnostics

AI is being applied across several stages of PTC management, primarily to improve diagnostic accuracy, refine risk stratification, and reduce rates of overtreatment. In the field of cytological and histological analysis, AI is revolutionizing the microscopic analysis of fine needle aspiration biopsy (FNA) and surgical samples [119]. Digital images of FNA slides help classify the sample with greater speed and consistency than manual evaluation which is crucial for reducing “indeterminate” results. On surgical specimens, algorithms can automatically identify tumor borders, papillary nuclear features (crucial for PTC diagnosis), and assess prognostic parameters like capsular or vascular invasion. The most sophisticated application of AI involves integrating complex data such as clinical, radiological, pathological, and molecular data (BRAF, RAS, TERT mutations, miRNA profiles) [120] to create robust predictive models that can forecast, with high accuracy, the probability of a tumor being aggressive, developing RAI resistance, or recurring years after surgery. This enables personalized therapeutic management from the outset (e.g., directing patients with high molecular risk toward more extensive thyroidectomy or more intense surveillance) [121].

### 8.7. The Liquid Biopsy Revolution in Advanced Cancer

A revolutionary advance in this field is the liquid biopsy, which offers a non-invasive alternative to traditional biopsy methods [122]. This technique relies on the analysis of circulating tumor DNA (ctDNA) and other plasma biomarkers, providing valuable real-time information on tumor aggressiveness and treatment response. Recent studies have shown that detecting the BRAF-V600E mutation in ctDNA correlates with PTC progression and therapeutic response [123,124], making liquid biopsy a promising tool in the management of advanced cases [125]. High-sensitivity techniques, such as microfluidic digital PCR and COLD-PCR (Co-amplification at Lower Denaturation temperature PCR), have further enhanced the ability to detect minimal quantities of genetic mutations in small plasma samples [126]. Integrating ctDNA analysis with the study of circulating tumor cells (CTCs) and ectosomes expands the diagnostic scope of liquid biopsy, allowing for continuous, dynamic monitoring of therapeutic efficacy and disease progression in thyroid cancer patients [127]. These advanced approaches allow for the definition of distinct molecular ATC subsets, essential for personalized therapeutic choice and prognostic evaluation.

## 9. Therapeutic Options in Thyroid Cancer

In the management of differentiated thyroid carcinoma (DTC), conventional treatments, including surgery, Radioactive Iodine (I^131^-RAI) therapy, and levothyroxine suppressive therapy, form the primary therapeutic pillars [128].

Specifically, total thyroidectomy is generally performed for PTC tumors exceeding 1 cm, though a partial lobectomy may suffice for microcarcinomas or low-risk forms, while FTC often requires only lobectomy for minimally invasive, non-angioinvasive forms, or total thyroidectomy followed by RAI for widely invasive or metastatic disease. Surgery remains the primary treatment in ATC when a complete resection is feasible. The goal is the removal of all macroscopic tumor residual; cytoreduction without complete resection is not recommended. For macroscopic extrathyroidal extension, total thyroidectomy may be indicated if technically feasible. The most effective treatment strategy is a multimodal approach combining surgery, radiotherapy, and systemic therapy. The use of bimodal or trimodal therapy has been shown to improve overall survival, with treatment choices individualized based on the patient’s performance status, tumor extent, and goals [129,130].

RAI is fundamental for intermediate-to high-risk patients to eliminate residual tissue and micro-metastases, but it is limited by side effects like salivary dysfunction and a small risk of secondary malignancies. Furthermore, levothyroxine is crucial for TSH suppression, lowering recurrence risk, but its use requires balancing benefits against the risk of cardiovascular side effects in vulnerable patients [131]. Despite their efficacy, these conventional strategies face significant limitations in patients who develop metastatic, dedifferentiated, or Radioiodine-Refractory (RAI-R) disease, a challenge that is driving a shift toward a precision medicine approach. RAI resistance is often linked to genetic mutations, notably BRAF-V600E and TERT promoter alterations, which compromise the tumor’s ability to concentrate iodine by reducing the expression of the Sodium-Iodide Symporter (NIS). The aggressive, refractory phenotype is further exacerbated by the co-occurrence of these mutations with alterations in genes like *TP53*, *PIK3CA*, and *AKT*, which synergistically activate the MAPK and PI3K/AKT/mTOR signaling pathways.

### 9.1. Targeted Therapeutic Strategies

The treatment landscape for advanced thyroid carcinoma has been revolutionized by the identification of molecular drivers, paving the way for highly effective targeted drug development. This precision approach utilizes both highly selective inhibitors and broader Multikinase Inhibitors (MKIs) [132].

**Figure 2:**
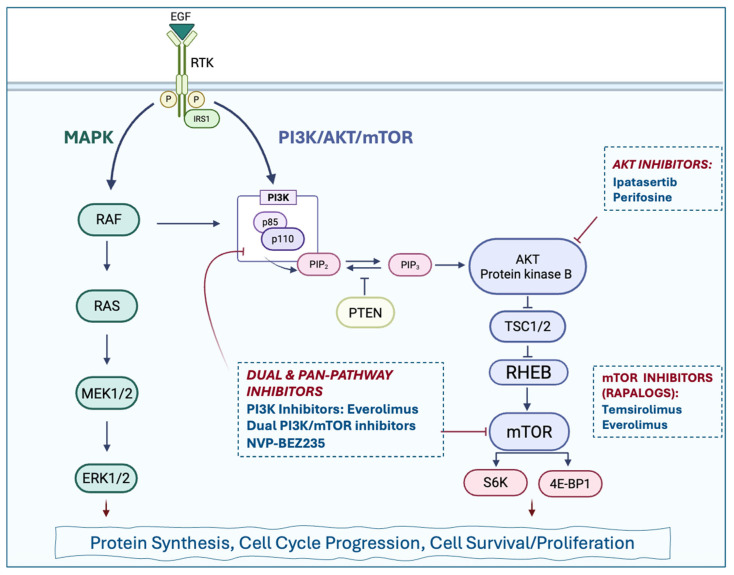
TK pathways (MAPK: mitogen activated tirosine kinase and PI3K/AKT/mTOR) activated by EGF. Oncogenic alterations or inactivation of PTEN cause the «always on state». Small-molecule inhibitors have been developed to directly block the hyperactive components (PI3K, AKT, mTOR) of the pathway.

Targeted agents primarily focus on three critical cancer signaling cascades. Firstly, the PI3K/AKT/mTOR Pathway is a key target in advanced non-medullary thyroid carcinomas (NMTCs), with agents showing clinical benefit including the mTOR inhibitors [133] like Everolimus and Temsirolimus, and the AKT inhibitors such as Perifosine and Ipatasertib. Secondly, the MAPK/ERK pathway is critical in many advanced papillary and follicular cancers. Future efforts in follicular thyroid cancer (FTC) specifically focus on developing inhibitors for the RAS pathway, while other advanced diseases benefit from therapies targeting downstream components of this axis. In *BRAF*-mutated tumors, this includes the use of BRAF inhibitors such as Vemurafenib, Dabrafenib, and Encorafenib, often paired with the MEK inhibitor Trametinib, to block the growth signal transmission [134]. Selective inhibitors represent a major advance because they are designed to target a single, specific oncogene, resulting in higher potency and fewer off-target side effects compared to MKIs. Figure 2 shows specific inhibitors of the above pathways.

The successful development of highly selective RET inhibitors—Selpercatinib [135] and Pralsetinib [136] has significantly advanced the treatment of Medullary Thyroid Carcinoma (MTC). Similarly, the initial identification of fusion drivers like STRN-ALK is paramount, as these targets can transform the prognosis of a disease notoriously resistant to standard therapies. ALK inhibitors such as Alectinib, Crizotinib, and Lorlatinib are highly effective in patients with rare *ALK*-rearranged ATC [137], where clinical data, though limited, shows rapid and durable clinical responses. In contrast, MKIs are systemic therapies often necessary for Radioactive Iodine-Refractory (RAI-R) Differentiated Thyroid Cancer (DTC) that is showing systemic or multifocal progression, where active surveillance is no longer appropriate. MKIs, such as Lenvatinib, Sorafenib [138] and Cabozantinib [139] target multiple Receptor Tyrosine Kinases (RTKs). These drugs target kinases involved in promoting new blood vessel formation, specifically the Vascular Endothelial Growth Factor Receptors (VEGFR), in addition to oncogenic drivers like RET. Finally, beyond these primary agents, future efforts for advanced thyroid cancers also include therapies targeting NTRK fusions and, in selected cases, immunotherapy with agents such as the anti-PD-1 antibody, Pembrolizumab (Table 4).

By disrupting these crucial signaling pathways, TKIs prevent cancer cells from receiving the continuous growth and division signals, thereby slowing tumor progression and offering an effective alternative systemic treatment for patients with RAI-refractory disease [140].

### 9.2. Immunotherapy

The development of combined strategies that modulate the immune microenvironment and enhance the response to immune check point inhibitors (ICIs) is therefore crucial for aggressive cancers (ATC and OCT). Emerging approaches, including CAR-T cell therapy, currently in Phase I trials, could represent a further significant step forward in treating highly aggressive malignancies [141,142]. Clinical trials with Spartalizumab (anti-PD-1) showed an overall response rate (ORR) of 19%, with responses observed primarily in PD-L1-positive patients. In cohorts with 50% PD-L1 expression, the response rate reached 35%, though patients with PD-L1 < 1% showed no response. Pembrolizumab showed similar results in clinical trials, with a 25% response rate and a median duration of response of 7.3 months in responders. These results suggest that immunotherapy alone is effective only in a limited fraction of patients, highlighting the need for strategic combinations. Recent studies have combined ICIs with targeted therapies such as ICIs + BRAF Inhibitors (vemurafenib/cobimetinib) (for BRAF mutant patients) have shown response rates up to 71% with a median overall survival of 18.23 months [143]. This demonstrates the superior efficacy of the multimodal ICI-targeted therapy approach. Combinations of ICIs with multikinase inhibitors (MKIs) like lenvatinib have also yielded promising results [144], with complete remissions observed in 66% of patients in retrospective analyses, showing a median progression-free survival (PFS) of 16.8 months and a median overall survival (OS) of 17.3 months. In the case of aggressive OTC characterized by an immune-depleted microenvironment associated with global LOH, the marked PD-L1 expression suggests that therapies targeting the PD-1/PD-L1 axis could potentially re-engage the immune system, despite the low overall T-cell infiltration. Given the dual nature of genomic instability and a dysfunctional TME in “cold” tumors, combinations of therapies, such as tyrosine kinase inhibitors (TKIs) with ICI may enhance T-cell infiltration and overcome the immunosuppressive barrier.

## 10. Conclusions and Future Perspectives

The present review highlights that thyroid cancer management is undergoing a significant transformation, moving from a predominantly morphological assessment toward a precision oncology approach driven by molecular diagnostics. The review’s core discussion points center on the profound impact of genetic drivers and the evolving classification system on risk stratification and personalized therapy. The rising incidence of thyroid cancer, largely attributed to enhanced detection, coexists with a recognition of increasing biological heterogeneity, demanding a more nuanced classification. The adoption of the WHO 5th Classification (2022) [5], with its explicit incorporation of molecular findings and the introduction of entities like DHGTC, is a crucial step. This system acknowledges that not all “differentiated” tumors are indolent, fundamentally changing how risk is assessed. Molecularly, thyroid cancer is a disease of aberrant signaling, primarily through the MAPK/ERK and PI3K/AKT/mTOR pathways [14,16]. The distinction between BRAF-like (more aggressive, less radioiodine-avid) and RAS-like (more indolent, follicular-patterned) tumors is now central to clinical decision-making. However, the most compelling finding is the synergistic role of the TERT promoter mutation in amplifying aggression, especially when co-occurring with BRAF-V600E [8].This molecular pairing serves as a powerful biomarker for the highest-risk subset, bridging the gap between differentiated and poorly differentiated disease. The presence of mutations like DAXX and EIF1AX in PDTC and ATC further supports the hypothesis of an evolutionary de-differentiation continuum [11,13]. Diagnostic advancements, particularly the refined use of molecular testing on indeterminate FNA samples, are critical for minimizing unnecessary diagnostic surgeries. The emerging role of AI in integrating complex clinical, radiological, pathological, and molecular data holds the potential to significantly improve diagnostic consistency and predictive accuracy. The goal of modern management is personalized therapy, using molecular and AI-driven risk stratification to guide the extent of surgery and the use of systemic treatments, thereby optimizing outcomes while minimizing patient morbidity [145]. In conclusion, the review aims to move beyond general knowledge to an individualized, molecular-driven approach to thyroid cancer management. Future research must focus on developing effective systemic therapies for the BRAF/TERT co-mutated and advanced ATC groups. This includes advancing clinical trials for BRAF, and PI3K pathway inhibitors and exploring novel strategies that overcome the mechanisms of RAI resistance. Deeper investigation into the TME, particularly the impact of TERT hyperactivation on immune evasion (e.g., PD-L1 expression and immune cell infiltration), is essential. This could pave the way for effective immuno-oncology strategies in poorly differentiated and high-grade thyroid cancers.

## Figures and Tables

**Table 1:** Classification by Cell of Origin (according to WHO 2022).

Category	Follicular Derivation
Benign Tumors	Follicular Adenoma, Oncocytic Adenoma, Follicular Adenoma with Papillary Architecture
Low-risk Neoplasms	Thyroid Tumor of Uncertain Malignant Potential (TTUMP), Hyalinizing Trabecular Tumor
Malignant Neoplasms	
Low-Grade	Low mitotic count, necrosis and Ki-67 index
High-Grade	High mitotic count, necrosis and Ki-67 index
	Differentiated High-Grade Thyroid Carcinoma (DHGTC) including: Papillary Thyroid Carcinoma (PTC) (various subtypes: classic, tall cell, hobnail, solid variant-SVPTC), Follicular Thyroid Carcinoma (FTC), Oncocytic Carcinoma (OC), Poorly Differentiated Thyroid Carcinoma (PDTC), Anaplastic Thyroid Carcinoma (ATC) (now includes the squamous cell subtype)
	**C-cell derived carcinoma**
	Medullary Thyroid Carcinoma (MTC)

**Table 2:** Mutations in PI3K/AKT/mTOR Pathway and in its regulator p53.

Component	Alteration Type	Effect	Thyroid Cancer Subtypes
*PI3K* (specifically the p110 a catalytic subunit, encoded by *PIK3CA* gene)	Activating Mutations, Gene Amplification	Direct activation of the pathway, leading to increased production of the second messenger PIP-3.	FTC, PDTC, ATC
*PTEN* (Phosphatase and Tensin Homolog)	Activating Mutations, Gene Amplification	Loss of this tumor suppressor, which normally inhibits the pathway by dephosphorylating *PIP-3*. This leads to sustained pathway activation.	FTC, PDTC, ATC
*AKT1*	Gene Amplification, Activating Mutations	Increased or hyperactive downstream signaling	ATC
*RAS* family genes (upstream of *PI3K*)	Activating Mutations	Can activate the *PI3K/AKT/mTOR* pathway (as well as the MAPK pathway), frequently co-occurring with other mutations in advanced disease.	FTC, PDTC, ATC
p53 (encoded by *TP53*)	Loss-of-Function Mutations, Loss of Expression	Loss of this major tumor suppressor, which normally negatively regulates the pathway (e.g., by transcriptionally upregulating *PTEN*).	PDTC, ATC (most frequent and critical in ATC), FTC

**Table 3:** MicroRNAs in thyroid cancer progression and metastasis.

miRNA	Regulation in Cancer	Functional Role	Aggressive Features Promoted	Primary References
miR-146b	Up-regulated	OncomiR. Promotes cell proliferation, survival, and inhibits differentiation. Targets tumor suppressors like PTEN and RARbeta (Retinoic Acid Receptor beta).	Strongly associated with: Lymph Node Metastasis (LNM), distant metastasis, higher risk of recurrence, and the *BRAF-V600E* mutation in PTC.	He et al., 2005 [26] Chou et al., 2013 [27]
miR-221/-222	Up-regulated	OncomiRs. Promote cell proliferation and decrease differentiation. Target the tumor suppressor *p27* (cyclin-dependent kinase inhibitor 1B, *CDKN1B*) and THRB (Thyroid Hormone Receptor Beta).	Strongly associated with: LNM, advanced TNM stage, and recurrence in PTC. Often overexpressed in aggressive PTC.	Visone et al., 2007 [28]
miR-21	Up-regulated	OncomiR. Anti-apoptotic and pro-proliferative effects. Targets tumor suppressors like *PTEN* and *PDCD4* (Programmed Cell Death 4), activating the *PI3K/AKT/mTOR* pathway.	Associated with: Poor differentiation, LNM, advanced TNM stage, and progression in PTC and Medullary Thyroid Cancer (MTC).	Visone et al., 2007 [28]
miR-451a	Down-regulated	Tumor Suppressor. Impairs proliferation and migration. Targets oncogenes like *MIF*, *c-MYC*, and *AKT1*, attenuating *AKT/mTOR* pathway activation.	Lower expression correlates with: Aggressive clinicopathological features in PTC (e.g., tall cell variant, advanced stage, extrathyroidal extension).	Minna et al., 2018 [29]
miR-145	Down-regulated	Tumor Suppressor. Inhibits cell growth, proliferation, and invasion. Its targets include genes in the *PI3K/AKT* pathway.	Associated with: Tumor growth and is generally under-expressed in PTC.	Zhang et al., 2020 [30]
miR-199a-3p/-5p	Down-regulated	Tumor Suppressor. Reduces MET and *mTOR* protein levels, impairing migration.	Associated with: Suppression of EMT and reduced LNM in PTC. Downregulated in tumors with LNM.	Peres et al., 2025 [31] Minna et al., 2014 [32]

**Table 4:** Multikinases and selective inhibitors used in thyroid cancers.

Inhibitor	Pathways Inhibited	Class
Ipatasertib	AKT (part of the PI3K/AKT/mTOR pathway)	AKT Inhibitor
Perifosine	AKT and mTOR signaling	Alkylphospholipid/AKT Inhibitor
Temsirolimus	mTOR (specifically mTORC1)	mTOR Inhibitor
Everolimus	mTOR (specifically mTORC1)	mTOR Inhibitor
Trametinib	MAPK/ERK pathway	MEK1 and MEK2 Inhibitors
Vemurafenib	BRAF V600E mutant	Selective BRAF Inhibitor
Dabrafenib	BRAF V600E and V600K mutant	Selective BRAF Inhibitor
Encorafenib	BRAF V600E, V600K, and V600D mutants	Selective BRAF Inhibitor
Selpercatinib	RET (Rearranged during Transfection)	Selective RET Inhibitor
Pralsetinib	RET (Rearranged during Transfection)	Selective RET Inhibitor
Alectinib	ALK (Anaplastic Lymphoma Kinase)	Selective ALK Inhibitor
Lenvatinib	VEGFR1-3, FGFR1-4, PDGFR alpha, KIT, RET	Multikinase Inhibitor
Sorafenib	RAF (C-RAF, B-RAF), VEGFR-2/3, PDGFR-beta,	Multikinase Inhibitor
Cabozantinib	MET, VEGFR2, RET, AXL (and others)	Multikinase Inhibitor
Crizotinib	ALK and c-MET	Multitarget TKI
Lorlatinib	ALK and ROS1 (Third-generation, designed to overcome resistance)	Multitarget TKI

## Data Availability

No new data were created or analyzed in this study. Data sharing is not applicable to this article.
